# Supplementary material for: Festuca coelestis Increases Drought Tolerance and Nitrogen Use via Nutrient Supply–Demand Relationship on the Qinghai-Tibet Plateau
Source: Plants (Basel). 2023 Apr 26;12(9):1773. doi: 10.3390/plants12091773 (PMC10181188; doi:10.3390/plants12091773)
Supplement: Supplementary file 1 [file plants-12-01773-s001.zip › plants-2245237-supplementary.pdf]

Supplementary Material

Supplement Figures:

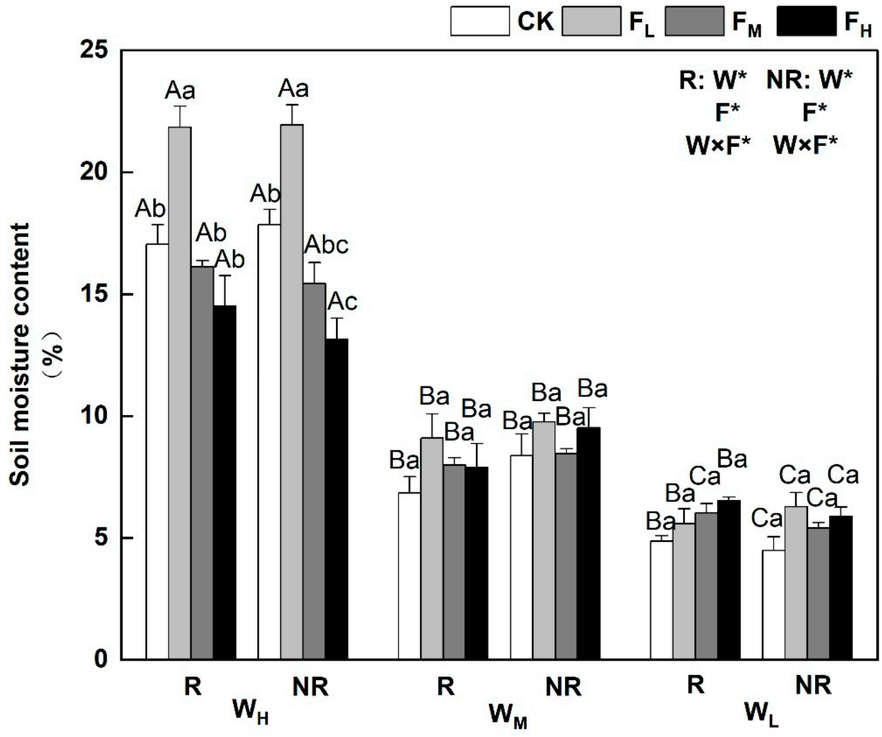

Figure S1. Changes of water content of *Festuca coelestis* under different water and fertilizer treatments

Notes: R and NR indicates rhizosphere soil and bulk soil. W;F;W×F indicates the effect of water, fertilizer and their interactions results of ANOVA ($P < 0.05$). Asterisk(*)and ns indicates significant difference and non-significant. The uppercase letters and lowercase letters represent the difference between water and fertilizer treatments. Asterisks before letters indicate significant differences between rhizosphere and bulk soils.

The effect of water and fertilizer on soil moisture content varied, and the rhizosphere and bulk soils showed same trend with no significant difference. Fertility effect was significant in the case of W_H, and reduced along with the decrease of moisture content. To be specific, the soil moisture contents of rhizosphere and bulk soils under W_H decreased as the fertility increased, and reached the highest points in the case of low fertilizer (F_L) treatment, which were 28.2% and 22.9% higher than those of CK, respectively ($P<0.05$). The soil moisture contents of *Festuca coelestis* under W_M and W_L were less affected by fertility.

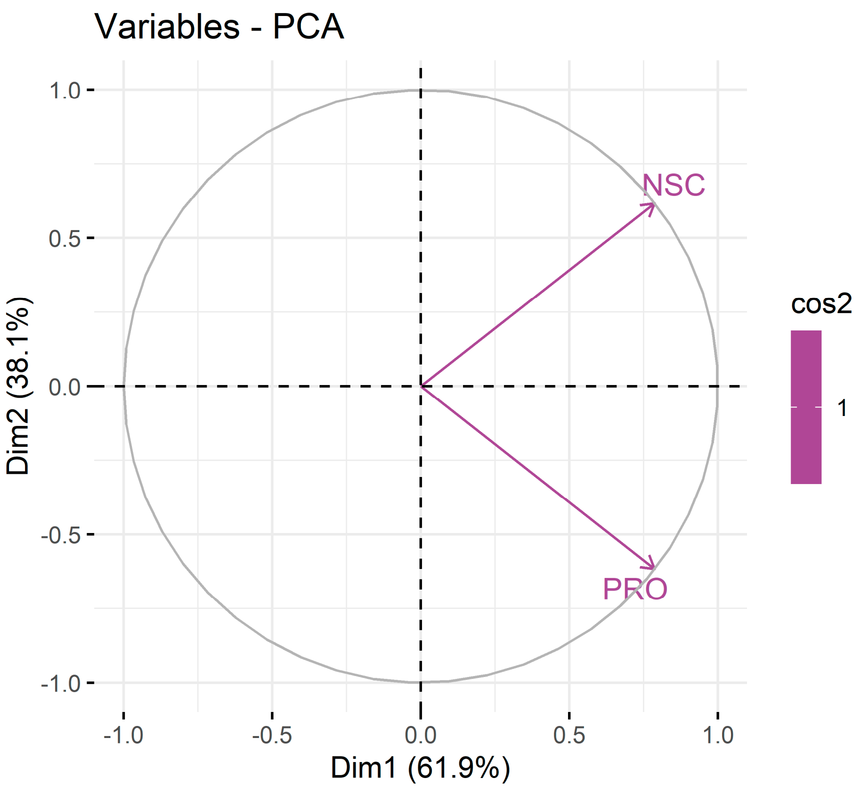

Figure S2 PCA can transform multiple factor variables of similar categories into a set of variables, here, converting "NSC" and "PRO" to "Physiological traits". subsequently, that will be used to construct SEM.

Notes: NSC (Non-structural carbohydrates); PRO (proline).
